# 

**Published:** 1865-11

**Authors:** 


Volume XXII. Number 11.

### THE CHICAGO

### MEDICAL JOURNAL

DE LASKIE MILLER, M. D.,

EPHRAIM INGALS, M. D.,

Editors and Proprietors.

NOVEMBER, 1865.

CHICAGO:

J. C. W. BAILEY, BOOK AND JOB PRINTER, 164 CLARK STREET.

1865.

